# Sustainable management of tick infestations in cattle: a tropical perspective

**DOI:** 10.1186/s13071-025-06684-4

**Published:** 2025-02-20

**Authors:** Eyabana Mollong, Marius Lébri, Carine Marie-Magdeleine, Stéphanie Marianne Lagou, Michel Naves, Jean-Christophe Bambou

**Affiliations:** 1 Département de Zoologie Biologie Animale, Laboratoire d’Entomologie Appliquée, Université de Lomé, Lomé, Togo; 2 Centre de Recherche en Écologie, Université Nangui Abrogoua, Abidjan, Côte d’Ivoire; 3 INRAE, ASSET, 97170 Petit-Bourg, Guadeloupe France; 4 UFR Agroforesterie, Université Jean Lourougnon Guédé Daloa, Daloa, Côte d’Ivoire

**Keywords:** Tick, Cattle, Sustainable management, Ethnoveterinary practices, Tropical regions

## Abstract

The increasing challenge of tick infestations in cattle production systems calls for innovative and sustainable solutions. This article explores the diverse ethnoveterinary practices and livestock farming systems prevalent in tropical regions, capitalizing on the characteristic biodiversity, to provide valuable insights and lessons for tick management at a global scale. Additionally, leveraging the genetic diversity of indigenous cattle breeds presents a promising approach for reducing reliance on chemical acaricides and fostering eco-friendly practices. While genomic selection is the cornerstone for addressing the polygenic nature of tick resistance, complementary genomic tools such as marker-assisted selection (MAS) can still play a valuable role. Integrating MAS and genomic tools could facilitate the development of tick-resistant cattle breeds, benefiting both tropical and temperate regions. Addressing challenges such as limited genomic resources, region-specific phenotyping methods, and the need for robust data infrastructure is essential for achieving tick management through genetic selection. Thus, tropical countries can contribute not only to their own livestock agriculture but also to global efforts in sustainable tick management. While some integrated approaches exist, further research is needed to objectively evaluate and optimize the combination of these different management strategies to effectively address tick challenges.

**Graphical Abstract:**

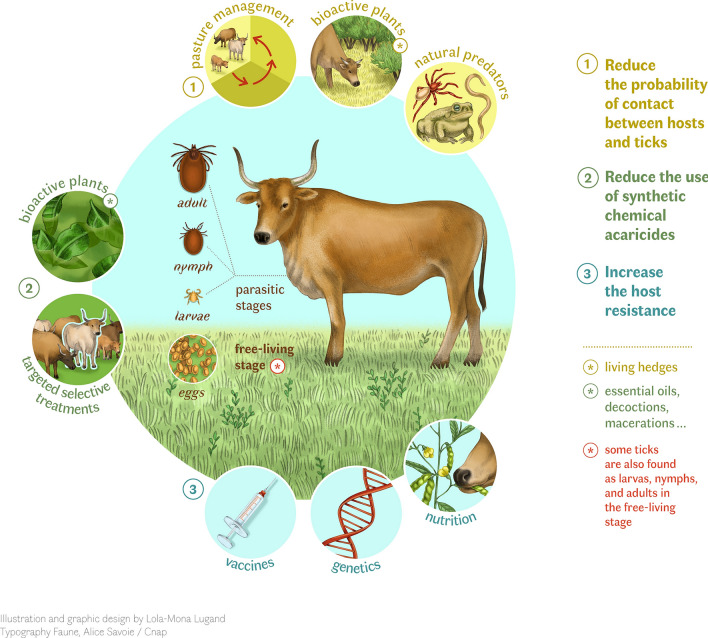

## Background

The agricultural potential of tropical countries is considerable given their rich biophysical diversity and favorable climatic conditions. Compared with temperate regions of the world, this high richness is described as the well-known “latitudinal gradient diversity.” Whatever the mechanism involved, evolutionary (i.e., greater rates of speciation coupled with lower rates of extinction) and/or ecological (i.e., a larger number of individuals supported by greater biomass productivity) events are lengthy processes that have occurred over a timescale of the order of millions of years [1, 2]. According to reports by the Intergovernmental Panel on Climate Change (IPCC), current global changes are occurring at a significantly faster pace than the natural climate cycle. It seems very likely that climate change has started to deeply impact the latitudinal diversity gradient, the most extensive and important biodiversity pattern on Earth. Thus, the consequences of this climatic change on species interrelationships, such as host‒parasite interactions, are among the most pressing issues facing agriculture today. Indeed, the climatic conditions in the tropics, along with their projected evolution, are particularly favorable for the development of numerous vector-borne and non-vector-borne infectious diseases, affecting humans, animals, and plant agro-resources [3, 4].

Infestations by ectoparasites, notably ticks, are among the most significant health problems affecting ruminant production in the tropics [5]. The direct impact of ticks on animal health and welfare is significant and strongly affects animal performance. Direct effects of ticks include animal stress, hide loss and udder injuries, reduction of milk production, and calf and adult animal mortality. Ticks are not only major obstacles to the success of ruminant breeding, posing a threat to food security and the livelihoods of local populations, but they also represent a high animal and human health risk, as in sub-Saharan African countries [6]. Ticks are vectors of various zoonotic pathogens, some of which are emerging diseases with a high risk for human health, particularly in populations suffering from malnutrition and, therefore, immunodepression (e.g., babesiosis, rickettsiosis, and borreliosis). In temperate regions, the escalating incidence of tick infestation risks outpacing the adaptive capacity of breeding systems unless proactive anticipatory strategies are implemented [7].

**Fig. 1:**
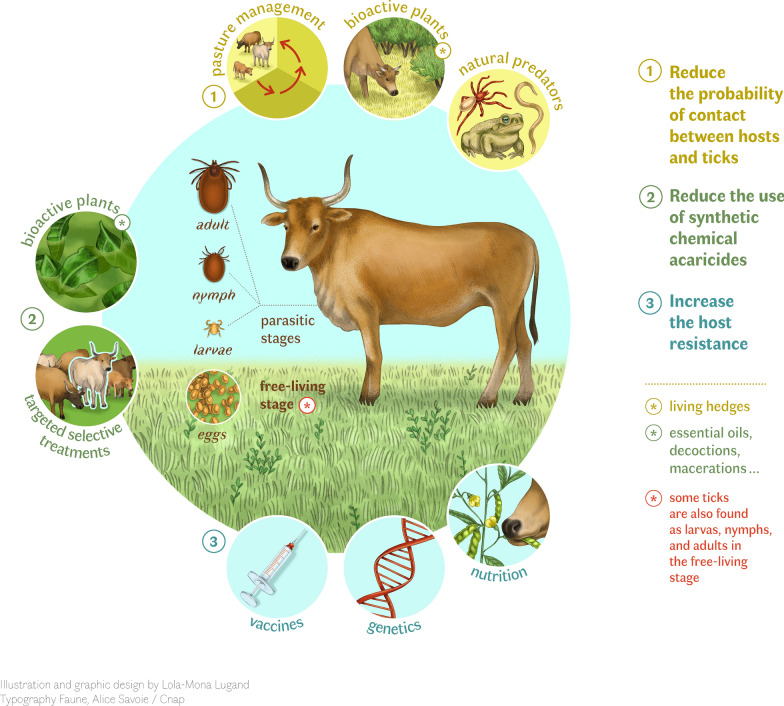
Conceptual framework of sustainable management of tick infestations

Regardless of the region of the world, the classical method of controlling tick infestation is the treatment of cattle with synthetic acaricide molecules. However, this method is not sustainable because of the increasing resistance of different ectoparasite populations to the acaricides at all latitudes, as well as the growing demand for animal products without veterinary drug residues and the desire to reduce deleterious effects on the environment [8]. Here, we review the extensive range of tick management strategies used in tropical countries, from conventional control methods to traditional and indigenous practices. This diversity in approaches likely reflects both the high incidence of tick infestations in tropical climates and the wide diversity of livestock farming systems, which range from intensive commercial farms to smallholder and subsistence-based farming systems. Additionally, we discuss the genetic diversity of the tropical cattle breeds, as reservoirs of genotypes resistant to tick infestations, highlighting their potential role in sustainable control practices. We propose a conceptual framework based on three key axes: (i) reduce host–tick contact, (ii) reduce reliance on synthetic chemical acaricides, and (iii) increase host resistance to tick infestations (Fig. 1). This generic integrated approach aims to promote more sustainable, environmentally friendly tick management strategies. Our aim is to demonstrate that tropical countries offer valuable insights and lessons that need to be studied more closely to meet the challenge of tick management on a global scale.

## Major ethnoveterinary practices and livestock farming systems for tick management: from tradition to knowledge

Livestock farming systems in the tropics are characterized by diverse production practices and livestock species, which can be categorized as extensive grazing systems, mixed crop‒livestock systems, agro-pastoral systems, and intensive production systems [9]. The smallholder farming systems are often based on traditional practices where animal facilities are usually open or partially open and surrounded by hedges made of posts or trees and shrubs. In these systems, ethnoveterinary practices play a crucial role in livestock health management, partly because medicinal plants are more accessible and affordable than synthetic veterinary drugs, and also because traditional practices are deeply rooted in the traditions of local communities. These practices have proven effective for addressing numerous livestock health problems [10]. Moreover, the range of grazing management practices is particularly diverse, with wide botanical diversity, including forages with acaricidal effects on ticks in the environment.

### Direct on-animal practices

In the past, the mere sight of ticks on their animals prompted smallholder farmers to pluck them off one by one to reduce the suffering of the animals, followed by crushing or burning them. This traditional method is still prevalent on many smallholder farms in Africa and has been documented in regions such as South Africa’s Eastern Cape Province [11, 12]. Unfortunately, pulling ticks fixed in the peri-anal area and scrotum can cause injury and defensive reactions by the animal. Moreover, the high parasitic load of *Rhipicephalus* (*Boophilus*) *microplus* makes this task cumbersome, and the long rostrum of *Amblyomma variegatum* can remain embedded in the animal's skin, leading to infections and abscesses [13]. Other farmers have resorted to washing the animals with salt water or smearing them with used motor oil, practices documented in South Africa and Burkina Faso [14–17]. These practices differ across regions, frequently reflecting traditional knowledge that has been transmitted through generations. This also applies to plant-based management strategies, whose phytosanitary use is rooted in empirical observations of certain plants that offer superior protection against tick infestations compared with others [18]. At present, numerous studies worldwide have demonstrated that bioactive plants rich in natural chemical groups such as alkaloids, tannins, flavonoids, saponins, quinone- based compounds (coumarin), sterols, and triterpenes can be utilized as alternatives to synthetic acaricides, with the synergistic action of different molecules [19–23]. Their effectiveness has been documented across diverse regions, including Africa, Asia, and Latin America, showcasing a global interest in leveraging the synergistic actions of plant-derived molecules for eco-friendly tick management. Essential oils are a type of biopesticide that consist of secondary metabolites produced by plants as a defense mechanism against phytophagous pests [24]. These oils find application in controlling ectoparasites in livestock. However, the extraction process and volatility of the oils necessitate appropriate techniques and formulations before field application. For instance, *Azadirachta indica* (neem) seed extract, commonly found in tropical regions such as India, Africa, and Southeast Asia, has demonstrated acaricidal activity, with neem oil formulations showing high mortality rates in *Ixodes* and *Rhipicephalus* spp. [23]. Traditional smallholder farmers often use extracts or decoctions from plants to prepare natural solutions with repellent or insecticidal properties against ectoparasites. These plant extracts are used for immersion baths or spraying, which allows more targeted application of the solution. Therefore, phytochemical studies employing various extraction methods (e.g., decoction, maceration, infusion, and hydrodistillation) should identify plants with the highest content of bioactive molecules that induce effective acaricidal activity. A notable case is the use of *Tephrosia vogelii*, a plant native to tropical Africa, whose leaf extracts have traditionally been used as an acaricide, the decoctions being applied topically to livestock to manage tick infestations, due to the presence of rotenoids, compounds with potent insecticidal and acaricidal effects [25, 26]. Moreover, a promising yet underexplored strategy involves supplementing livestock feed with bioactive plants or their derived compounds, analogous to approaches used for managing endoparasite infections in small ruminants [27]. Similarly, nutritional manipulation of small ruminants has long been considered a tool for the control of gastrointestinal nematode (GIN) infections [28]. Numerous feeding trials with small ruminants have demonstrated that improved nutritional status can reduce production losses and mortality rates due to GIN infection [29, 30]. The respective roles of protein and energy supplies in improving host resistance to GIN infection remain unclear. For tick infestations, nutritional strategies have been poorly investigated. A previous study demonstrated that *Amblyomma americanum* burden significantly influences various aspects of growth and metabolic function in Angus cross steers, with the detrimental effects being intensified under suboptimal nutritional conditions, suggesting that improving nutritional status could attenuate the negative impacts of infestations [31]. Similarly, following experimental *R.* (*B.*) *microplus* larval infestations, the percentage yield of mature ticks was approximately three times greater in animals fed a low-quality diet than in those fed high-quality feed, highlighting the pronounced influence of nutrition on host resistance to tick development [32]. However, it is important to stress the complexity of this approach, due to the interactions between nutrition, host genotypes, tick species and infestation dynamics, and production systems and environmental conditions (temperate, subtropical, tropical).

To minimize acaricide use and reduce the risk of resistance development in tick populations, selective treatment, which targets only animals with a pre-established tick load rather than treating the entire herd at fixed intervals, has been recommended as a more sustainable strategy [33, 34]. Although this approach is widely accepted for reducing the selection pressure for resistance genes in gastrointestinal parasites, its effectiveness for tick infestations has been questioned [35]. A recent study challenged the effectiveness of this approach in managing tick populations [36]. However, the ecological impact of the increased number of acaricide treatments required when treating the entire herd at fixed intervals must be considered for a comprehensive comparison of the two strategies. A holistic evaluation should account for the potential environmental consequences of widespread chemical use alongside the benefits of reduced infestation levels. Moreover, effective management of acaricide practices in African countries requires a multidisciplinary approach that integrates social science knowledge, veterinary expertise, and community strategies. Mutavi et al. [37] highlight the critical need for targeted education and adherence to best practice in order to optimize the use of acaricides and mitigate the development of tick resistance to the acaricides.

### Indirect on-pasture practices

Tick control aims primarily to mitigate both the direct losses caused by ticks and the losses associated with diseases transmitted by these ticks [38, 39]. Consequently, pasture management is a fundamental aspect of sustainable control strategies designed to disrupt the developmental cycle of ticks. Targeted interventions can be implemented to interfere with tick proliferation within the biotope [40]. These interventions include, among others, the use of bioactive plants, pasture rotation, and the promotion of tick predators.

Local bioactive plants have been studied for their capacity to control ticks in tropical and subtropical regions [41]. Plants with acaricidal or tick repellent properties are used by traditional smallholder livestock farmers as separation or corridor hedges in large pasture areas [42] and near their housing for protection [13]. The bioactive compounds released by various organs of these plants (i.e., roots, bark, leaves, fruits, or seeds) can significantly disrupt the development of ticks and other parasites in pastures over an extended period. One such plant is *Clausena anisata* (Willd.) Hook. f. ex-Benth, a member of the family Rutaceae whose acaricidal and tick repellent activities have been demonstrated in Togo [43, 44]. The use of forage plants with bioactive properties, such as *Stylosanthes* spp., *Melinis minutiflora*, and *Andropogon gayanus*, have been studied in regions including South America (Brazil and Colombia), Puerto Rico, India, and Australia. These plants have shown potential for trapping or repelling tick larvae and adults in pastures, thereby decreasing overall tick loads [42, 45, 46]. Pasture management practices additionally contribute to local plant biodiversity conservation. Biocidal plant corridor hedges in pastures also play a role in preserving and restoring faunal biodiversity, providing habitats for various tick predators (Table 1). This integrated synergistic action, as already observed by Mishra et al. [46], contributes to the conservation of different environmental compartments.

**Table 1:** Tick parasites, parasitoids, and predators throughout the world

Taxonomic class (common name)	Different tick enemy species	Tick species	Geographical regions	Mode of impact on host	References
Sordariomycetes (fungi)	*Beauveria bassiana*, *Lecanicillium* (*Verticillium*)* lecanii* *Metarhizium anisopliae*	*Amblyomma variegatum* *Hyalomma* sp. *Ixodes scapularis* *Rhipicephalus* (*Boophilus*) *microplus*, *R. appendiculatus*, *R. sanguineus*	Tropical and subtropical regions (Africa, Asia, Americas)	Fungal infection of ticks, leading to mortality of larvae and adults	[72]
Nematoda (nematodes)	*Steinernema carpocapsae* *Steinernema glaseri* *Steinernema feltiae* *Heterorhabditis* sp.	*A. maculatum*, *A. americanum*, *H. dromedarii*, *H. excavatum*	Worldwide (except polar regions)	Nematodes infect tick larvae, causing death via parasitism	[52, 73]
Insecta (insects)	*Solenopsis invicta* *Solenopsis geminata* *Megaselia scalaris* *Tineola* sp. *Ixodiphagus hookeri*	*A. maculatum*, *A. americanum* *R.* (*Boophilus*)* microplus*, *A. variegatum* *Ixodes *sp*.,* *Dermacentor* sp., *A. variegatum*	Tropical, temperate (mainly in North and South America, Africa)	Feed on ticks by ants, spiders; parasitism by *Ixodiphagus* on tick larvae	[51] [58] [61, 74]
Arachnida (spiders)	*Schizocosa ocreata* *Dysdera murphyi*	*I. scapularis*, *I. persulcatus* *A. variegatum* *R. sanguineus*	Tropical, temperate	Feed on ticks, species like *Ixodiphagus* parasitize tick larvae	[75] [76]
Amphibia (amphibians)	*Bufo paracnemis*	*R. sanguineus* *A. cajennense*	South America	Larvae feed on tick larvae or nymph	[75]
Reptilia (reptiles)	*Tropidurus* sp.	*Ornithodoros amblus*	Tropical and subtropical regions (South America)	Feed on tick larvae or nymph	[75]
Aves (birds)	*Gallus gallus* *Meleagris gallopavo* *Buphagus africanus*, *Buphagus erythrorynchus* *Bubulcus ibis* *Numida meleagris*	*R. decoloratus*, *H. marginatum rufipes*, *R. evertsi evertsi* and *Otobius megnini* *I. scapularis* *Hyalomma* sp. and *Rhipicephalus* sp.	Worldwide (especially in Africa and Europe)	Feed on ticks	[63] [64] [65] [66]
Mammalia (mammals)	*Peromyscus leucopus*	*I. scapularis*	Temperate, subtropical regions	Feed on ticks	[77] [78]

This list is non-exhaustive

The cultivation of indigenous bioactive plants leverages biotechnological advancements to enable mass production of seeds and plants, facilitating the reforestation of areas surrounding livestock farming systems and grazing lands. Concurrently, a One Health approach is essential to assessing the safety of these plants for animals, humans, and ecosystems, considering their dual role as sources of medicinal compounds. Such research has the potential to substantially enrich tropical pharmacopoeia while promoting sustainable agricultural practices, enhancing biodiversity conservation, and fostering interconnected benefits between agriculture, human health, and ecosystem integrity.

Rotational grazing, on the other hand, involves removing or rotating animals from tick-infested pastures for a specific duration to starve the ticks, interrupting their life cycle and preventing contact with hosts. This method has been evaluated in various regions, including West and South Africa, as well as South America (Mexico, Venezuela), for its potential to control *Rhipicephalus* spp., *Hyalomma* spp., and *Amblyomma hebraeum* [47–49]. Experimental studies and modeling suggest it can be effective in disrupting tick infestation cycles. Moreover, when combined with acaricidal treatment, rotational grazing has been shown to effectively disrupt the infestation cycles of *A. variegatum* nymphs in Burkina Faso [14]. However, it may not be fully effective against adult ticks with longer cycles, such as species belonging to the genera *Amblyomma*, *Hyalomma*, and *Ixodes*. To address this issue, supplementary measures such as clearing and cultivating tick-infested areas, along with controlled burning during the dry season, can be utilized to disrupt the development of ticks with extended life cycles [50]. Therefore, an integrated approach that combines various methods, including pasture management, is essential for effective on-site tick control.

A promising biological control approach, which is still in the research phase, utilizes parasites, parasitoids, and predators that target ticks as hosts or prey. It effectively reduces tick populations and the transmission of zoonotic pathogens to animals and humans (Table 1). Tick predators are diverse and include approximately 850 animal species ranging from nematodes to mammals, including a large taxonomic class of insects [51, 52]. The fungi *Beauveria bassiana* and *Metarhizium anisopliae* have also been found to affect the ticks *Rhipicephalus appendiculatus* and *A. variegatum* [53–55]. In addition, two species of nematodes, *Steinernema glaseri* and *Steinernema feltiae*, are capable of killing 30–100% of engorged *Amblyomma maculatum* and *A. americanum* female ticks [56, 57]. The same tick species can be targeted by the ant *Solenopsis invicta* (Hymenoptera: Formicidae) depending on their habitat [58]. Barré et al. [59] demonstrated that *Solenopsis geminata* prey on *R.* (*B.*) *microplus* ticks and the engorged stages of *A. variegatum*. Additionally, *Ixodiphagus* wasps, belonging to the family Encyrtidae, act as hyperparasites of ticks, although their exact impact on reducing tick populations remains debated [60]. A study in Kenya reported a 51% parasitism rate of nymphal *A. variegatum* following the release of *Ixodiphagus hookeri* wasps over 1 year, leading to a significant reduction in tick infestations on cattle, with mean counts decreasing from 44 to two ticks per animal [61]. However, the literature remains scarce regarding the use of wasp as a potential means of biological control of ticks in cattle production systems, with limited studies addressing their feasibility and long-term efficacy in such contexts. Alternatively, ant species have demonstrated predatory behavior in tick laboratory settings [62]. However, this predatory ability against both hard and soft ticks requires confirmation under natural conditions. Due to their predatory behavior toward various arthropod populations, the effectiveness of birds such as the guinea fowl (*Numida meleagris*), partridges (*Perdix perdix*), and cattle egrets (*Bubulcus ibis*) in controlling tick populations has been explored in various studies [63–66]. Some studies have demonstrated the predatory behavior of some of these birds towards ticks but have failed to show a significant reduction in tick abundance, suggesting instead that they may serve as hosts for certain tick species. Indeed, cattle egrets have been shown to serves as hosts for immature *A. variegatum* in the eastern Caribbean, facilitating their migration between islands in the region. This highlights their contradictory role in both the potential control and dissemination of the tick [67]. The synergistic effects of integrating multiple strategies must be rigorously evaluated before implementation, with the goal of enhancing the conservation of plant and animal diversity while effectively controlling tick populations and mitigating the transmission of tick-borne diseases.

## Leveraging genetic diversity in tropical livestock for sustainable tick management

Exploiting host resistance to ticks is a promising and sustainable long-term strategy for developing effective management methods. In cattle, host resistance to ticks offers a cost-effective and long-term solution that requires no additional labor or resources [68]. While research currently focuses on new anti-tick vaccines and acaricides, enhancing host resistance has been largely overlooked. This is despite its potential for sustainable tick control, particularly in tropical breeds where resistance, which has low to moderate heritability (0.13–0.44), should not be underestimated [5]. The genetic diversity of cattle in tropical regions provides a valuable reservoir of adaptive genes, which could be crucial for identifying genetic resistance to ticks. Indigenous cattle breeds, such as the N'Dama in West Africa, Brahman and Sahiwal in India, and Creole and Romosinuano in Latin America, have co-evolved with their environments over centuries, developing natural resistance to ticks (Table 2). These native breeds have exhibited remarkable resistance to tick infestations and require minimal chemical intervention for tick control. Integrating these breeds into cattle production systems represents a promising strategy for reducing reliance on chemical acaricides and promoting eco-friendly practices. Furthermore, crossbreeding and within-breed selection programs have demonstrated success in enhancing cattle resistance to ticks [69]. Recent genome-wide association studies (GWAS) have identified genetic markers and genes associated with tick resistance in South African Nguni cattle and Argentine Creole cattle [70, 71]. In Nguni cattle, a genomic region on BTA17 has been associated with resistance to *Rhipicephalus evertsi evertsi* ticks, a species primarily distributed in Africa, highlighting the presence of the LRBA (LPS-responsive vesicle trafficking, BEACH [Beige and Chediak-Higashi domain], and anchor-containing) gene, which plays a role in immune effector molecule secretion. Additionally, genes such as small integral membrane protein 12 (*SMIM12*), Fer (fps/fes-related) tyrosine kinase (*FER*), and *LINGO2* (Leucine-rich repeat and Ig domain-containing 2) were identified for tick resistance, though their precise contributions to resistance or susceptibility remain unclear. In the study on Argentine Creole cattle, genes involved in immune response, blood coagulation, tissue regeneration, and protein phosphorylation were identified, including kallikrein (KLK), fibroblast growth factor 20 (FGF20), thymocyte-expressed molecule involved in selection (THEMIS), IQ motif-containing GTPase-activating protein 2 (IQGAP2), and nitric oxide synthase 2 (NOS2) (located on BTA18, 9, 10, and 19, respectively). Marker-assisted selection (MAS), introgression, and genomic selection offer exciting opportunities to select lines that inherit desirable production traits while simultaneously enhancing resistance to ticks. These genetic tools have the potential to accelerate the development of tick-resistant cattle breeds or lines, promoting sustainable tick management across both tropical and temperate agricultural systems. However, practical implementation in tropical regions faces several challenges. These include the limited availability of genomic resources tailored to the genetic diversity of tropical cattle breeds, the costs of genomic analysis, and the need for region-specific and scalable phenotyping methods. Furthermore, the absence of well-established historical reference populations complicates the application of genomic selection in tropical livestock.

**Table 2:** Cattle breeds resistant to tick infestation

Breeds	Subspecies	Countries/region of origin	Challenge	Tick species^a^	References
Argentine Creole		Argentine	Artificial infestation	*Rhipicephalus* (*Boophilus*)* microplus*	[79]
Indigenous Vechur	Indicus	India	Natural	*R.* (*Boophilus*) *annulatus* (43.4%), *H. bispinosa* (32.1%), *R. haemaphysaloides* (1.89%), *Amblyomma* sp. (1.89%), *R.* (*Boophilus*) *microplus* (15.09%), and *Rhipicephalus* sp. (5.66%)	[80]
Kasaragod Dwarf cattle	Indicus	India	Natural	*R.* (*Boophilus*) *annulatus* (43.40%), *H. bispinosa* (32.06%), *R. haemaphysaloides* (1.89%), *Amblyomma* sp. (1.89%), *R.* (*Boophilus*) *microplus* (15.09%), and *Rhipicephalus* sp. (5.66%)	[80]
Nguni cattle	Taurus	South Africa	Natural	*Amblyomma hebraeum*, *Rhipicephalus* (*Boophilus*) *decoloratus* and *Hyalomma* spp.	[81]
Bonsmara cattle	Taurus	South Africa	Natural	*A. hebraeum*	[82]
Afrikaner	Taurus	South Africa	Natural	*A. hebraeum*	[83]
Senepol	Taurus	West Indies	Artificial infestation	*R.* (*Boophilus*)* microplus*	[84]
Mbullu	Indicus	Tanzania	Natural	*Amblyomma* sp., *Rhipicephalus* sp. and *Hyalomma* sp.	[85]
Iringa red zebu	Indicus	Tanzania, Kenya	Natural	*Amblyomma* sp., *Rhipicephalus* sp., *Hyalomma* sp. and *Rhipicephalus* sp.	[85]
N'Dama cattle	Taurus	Guinea	Natural	*A. variegatum*	[86]
Brahman	Indicus	India	Artificial infestation	*R.* (*Boophilus*)* microplus*	[87]
Sahiwal	Indicus	India	Natural	*R. appendiculatus*	[87]
Ankole	Indicus	Eastern Africa	Natural	*R. appendiculatus*	[87]
Romosinuano	Taurus	Colombia	Natural	*R.* (*Boophilus*)* microplus*	[88]

This list is non-exhaustive

^a^Tick species: when available in the study, the relative abundance of each species is presented as a percentage

To harness the genetic diversity of tropical cattle for effective tick management, cost-effective and easily implementable phenotyping strategies must be developed to evaluate resistance across diverse breeds to various tick species [68]. Additionally, further research is needed to understand the underlying mechanisms of genetic resistance and to refine breeding strategies to increase tick resistance while maintaining productivity, thus ensuring sustainable outcomes in tropical livestock systems.

## Conclusions

The rich biophysical diversity and climatic conditions of tropical regions have fostered a wide array of ethnoveterinary practices and husbandry systems for tick management. These extensive practices, rooted in traditional knowledge, have proven to be valuable in combatting the health and economic challenges posed by tick infestations in livestock production. Among the most relevant and effective methods for tick management are bioactive plants with acaricidal or repellent properties. When strategically used in combination with pasture management and animal treatments, these plants can significantly reduce tick populations and minimize reliance on synthetic acaricides. However, it is important to consider these uses within integrated management strategies, where pasture management practices, such as rotational grazing, are used to disturb the tick life cycles, thereby reducing the overall infestation burden on cattle. Furthermore, the choice of indigenous cattle breeds known for their resistance to tick infestations, could also reduce the need for synthetic acaricides. The development of integrated tick management strategies requires the strategic combination of these different methods. This approach should be further refined through both conceptual and mathematical modeling to identify existing gaps that still need to be experimentally investigated. By recognizing the potential of tropical countries to offer valuable insights and solutions, we can foster international collaboration and knowledge exchange, benefiting livestock farming worldwide and promoting harmonious coexistence between agriculture and the environment.

## Data Availability

No datasets were generated or analyzed during the current study.
